# Evaluation of single nucleotide polymorphisms of *pvmdr1* and microsatellite genotype in *Plasmodiumvivax* isolates from Republic of Korea military personnel

**DOI:** 10.1186/s12936-015-0845-6

**Published:** 2015-09-04

**Authors:** Dong-Il Chung, Sookwan Jeong, Sylvatrie-Danne Dinzouna-Boutamba, Hye-Won Yang, Sang-Geon Yeo, Yeonchul Hong, Youn-Kyoung Goo

**Affiliations:** Department of Parasitology and Tropical Medicine, Kyungpook National University School of Medicine, 101 Dongin-2-ga, Joong-gu, Daegu, 70-422 Republic of Korea; Department of Medicine, Headquarters of Republic of Korea Army, Choongnam, Republic of Korea; College of Veterinary Medicine, Kyungpook National University, Daegu, 702-701 Republic of Korea

**Keywords:** *Plasmodium vivax*, Multidrug resistant-1 gene, Microsatellite marker, Genetic diversity

## Abstract

**Background:**

Chloroquine has been administered to the soldiers of the Republic of Korea as prophylaxis against vivax malaria. Recent increase in the number of chloroquine-resistant parasites has raised concern over the chemoprophylaxis and treatment of vivax malaria.

**Methods:**

To monitor the development of chloroquine-resistant parasites in the Republic of Korea, analyses of single nucleotide polymorphisms (SNPs) of *pvmdr1* and microsatellite markers were performed using samples collected from 55 South Korean soldiers infected with *Plasmodium vivax*.

**Results:**

Four SNPs, F1076L, T529, E1233, and S1358, were identified. Among these, S1358 was detected for the first time in Korea. The microsatellite-based study revealed higher genetic diversity in samples collected in 2012 than in 2011.

**Conclusions:**

Taken together, the results indicate that *P*. vivax with a newly identified SNP of *pvmdr1* has been introduced into the Korean *P. vivax* population. Therefore, continuous monitoring for chloroquine-resistant parasites is required for controlling vivax malaria in the Republic of Korea.

## Background

Malaria caused by *Plasmodium vivax* is the most common human malaria infection, affecting 40 % of the world’s population [1, 2]. In the Republic of Korea, vivax malaria had been successfully eliminated by the late 1970s by an effective World Health Organization (WHO) programme. However, this infectious disease has re-emerged, since a soldier was diagnosed with *P. vivax* infection in 1993 [3, 4]. Since then, vivax malaria has been the only type of malaria detected in the Republic of Korea, accounting for 18,052 cases reported from 1994 to 2013 [5].

Vivax malaria endemic regions in the Republic of Korea are concentrated near the demilitarized zone that separates South Korea from the Democratic People’s Republic of Korea (DPRK or North Korea). Thus, military personnel and residents living in the demilitarized zone are under high risk of contracting vivax malaria. Military personnel accounted for 25.3 % (1029/4063) of all malaria cases reported from 2008 to 2010 [6]. This percentage rose to 44.7 % (1811/4063) when military personnel diagnosed with vivax malaria following discharge from the service were counted [7]. In this regard, mass chemoprophylaxis using chloroquine and primaquine has been administered to soldiers since the year 1997 to control vivax malaria infection. For the chemoprophylaxis, 300 mg chloroquine is administrated weekly to military personnel from June to August (for 12 weeks), and then, 30 mg primaquine is administrated daily for 2 weeks.

Chloroquine has been used to not only kill *P. vivax* in asexual blood stages and gametocytes, but also to prevent the spread of malaria in low-risk areas. However, its massive use in the treatment of vivax malaria and continuing long-term chemoprophylaxis could facilitate the acquisition of resistance to chloroquine [8]. Since the first report of chloroquine-resistant *P. vivax* in 1989 in Papua New Guinea, the number of chloroquine-resistant cases has increased in several countries, including Indonesia, Southeast Asia, India, and Central and South America [9–15]. Although cases of chloroquine-resistant malaria infections have been confirmed recently, chloroquine is still used as the therapeutic and chemoprophylactic drug for *P. vivax* infections in the Republic of Korea [16]. Thus, the administration of chloroquine to soldiers stationed near the demilitarized zone has raised the concern of accelerating the development of drug-resistant *P. vivax*.

Monitoring the genetic polymorphism that confers chloroquine resistance to malaria provides useful information regarding the efficacy of drugs in treating malaria. However, compared to *P. falciparum*, previous studies using genetic markers for chloroquine-resistant *P. vivax* did not conclude a strong correlation between the genetic markers and chloroquine-resistant phenotype in *P. vivax*, because the molecular mechanisms of chloroquine resistance in *P. vivax* is still elusive [17]. Among the genetic markers for chloroquine-resistant *P. vivax*, the multidrug resistance-1 gene of *P. vivax* (*pvmdr1*) has been identified as a possible genetic marker of chloroquine resistance with in vitro characterization of isolates [18]. In Southeast Asia and Papua New Guinea, the Y976F mutation (TAC→TTC) in *pvmdr1* has been shown to be correlated with chloroquine resistance [19–21]. In addition, the association of severe malaria and expression levels of *pvmdr1* with chloroquine resistance was reported by showing 2.4-fold increase in *pvmdr1* expression levels in parasites from patients compared to the susceptible group of vivax malaria in the Brazilian Amazon [21]. This chloroquine resistance appears to be the result of a two-step mutation pathway, in which the F1076L mutation is followed by the Y976F mutation [22, 23]. The F1076L mutation is found in all Korean samples tested, and is unlikely to result in chloroquine treatment failure [24], while the Y976F mutation has not yet been reported in the Republic of Korea [25]. However, considering that the South Korean military has been performing mass chemoprophylaxis for more than 15 years, there likely is strong evolutionary pressure for selection of the double mutant.

Recently, the genetic diversity as well as intra- and inter-population relationships of *P. vivax* isolates obtained from the Republic of Korea (from 1994 to 2008) were analysed [26, 27]. By using microsatellite markers, this analysis provided an explanation for the genetic diversity observed among strains. In this study, the prevalence of five common non-synonymous single-nucleotide polymorphisms (SNPs) and four synonymous SNPs at the *pvmdr1* locus, including the Y976F and F1076L, was examined in 55 *P. vivax* isolates obtained from military vivax malaria patients who had taken chemoprophylaxis near the demilitarized zone of the Republic of Korea. The population structure of these isolates was analysed using the microsatellite method with 10 microsatellite markers.

## Methods

### Ethics statements

This study was approved by the ethics committee of the Army Forces Medical Command (Approval No. AFMC-13-IRB-053, July 2011). An approval form was used to obtain written informed consent from each participant and all participants provided their informed consent for collecting a 5-mL blood sample.

### Blood samples and DNA extraction

**Fig. 1:**
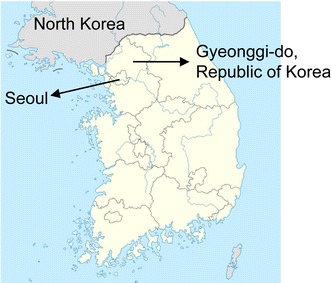
Map of Gyeonggi-do Province in the Republic of Korea: endemic regions of vivax malaria near the demilitarized zone in which samples were collected

A total of 55 blood samples were collected from patients infected with malaria and admitted to the Armed Forces Hospitals near the demilitarized zone located in northern Gyeonggi-do Province in the northwest region of the Republic of Korea in 2011 and 2012 (Fig. 1). The admission and clinical management of the patients were undertaken independently of this study. Aliquots (200 μL) of venous blood samples were stored at −20 °C in ethylenediaminetetraacetic acid (EDTA)-coated bottles until extracting genomic DNA using a QIAamp^®^ DNA Blood Mini Kit (Qiagen, USA) according to the manufacturer’s instructions.

### Identification of single nucleotide polymorphism (SNP) in the *pvmdr1* gene

The *pvmdr1* gene was amplified by nested PCR using *pvmdr1* gene specific primers [25, 28]. Amplification of *pvmdr1* gene fragments was performed applying a nested PCR approach and regents, primers, and cycling conditions as outlined in Table 1. The final PCR products were resolved by electrophoresis on a 1.5 % agarose gel stained with ethidium bromide, and visualized under ultraviolet illumination. The second PCR products were sequenced and the deduced amino acid sequences were compared to the amino acid sequence of *pvmdr1* from the Sal I strain of *P. vivax* (Salvador I, GenBank accession no. AY571984). The amino acid sequence alignment and analyses were performed using Clustal Omega [29].

**Table 1:** Primers and cycling condition of a nested PCR for amplifying *pvmdr1*

	Primer name	Sequence (5′–3′)	Annealing temperature (°C)	Size of PCR product (bp)
First	*pvmdr1* F1	TTGAACAAGAAGGGGACGTT	61	4290
	*pvmdr1* R1	CTTATATACGCCGTCCTGCAC		
Second	*pvmdr1* F2	CAGCCTGAAAGATTTAGAAGCCTT	58	539
	*pvmdr1* R2	CATCCACGTCCACAGTGGAAC		
	*pvmdr1* F3	GGATAGTCATGCCCCAGGATTG	62	604
	*pvmdr1* R3	CATCAACTTCCCGGCGTAGC		
	*pvmdr1* F7	GATGAGCCTGCTGATGCGATTCTAC	60	745
	*pvmdr1* R5	ATATACGCCGTCCTGCACCGAG		

### Analysis of ten microsatellite markers

To determine the relationships between the different *pvmdr1* genotypes of *P. vivax*, 10 microsatellite markers were typed for 28 samples collected in the year 2011 and 27 collected in the year 2012. The 10 microsatellite markers used for this assay were as follows: MS1, MS3, MS5, MS8, MS10, MS12, MS16, MS20, Msp1F7, and Pv3,27. The primer sets and amplification conditions used for the PCRs have been described elsewhere [30, 31]. The fluorophore-labelled PCR products were quantified using an Applied Biosystems 3730 DNA Analyzer with the GeneMapper software Version 4.0 (Applied Biosystems, USA). In order to reduce potential artifacts from background noise or stutter, an arbitrary fluorescent intensity threshold of 50 relative florescence units was applied for peak detection. All electropherogram traces were additionally inspected manually. For each isolate, at each locus, the predominant allele, the highest intensity peak and any additional alleles with a peak height of at least one-third of the height of the predominant allele were scored [32]. Genotyping success was defined as the presence of at least one allele at a given locus in a given sample.

### Population genetic analyses and statistical treatments

The major alleles of each locus were used for our population genetic analysis. The level of genetic diversity of the *P. vivax* population in Republic of Korea was assessed by allele number per locus (A) and expected heterozygosity (*He*). The *He* values for each locus were calculated using the formula *He* = [n/(n − 1)] [1 − ∑*p*_*i*_^2^], where n is the number of isolates examined and *p*_*i*_ is the frequency of the ith allele. The statistical significance of the differences in these values was evaluated by Welch’s *t* test.

Multilocus linkage disequilibrium (LD) was assessed using LIAN v3.6 based on the allelic data for the 10 microsatellite DNA loci [33]. This program computed the standardized index of association (*I*_A_^S^), a measure of genotype-wide linkage. The *P*-values were determined by a Monte Carlo simulation process, performing 100,000 iterations. Only those samples for which a complete set of microsatellite alleles were scored were used for this analysis. Additionally, the multilocus genotypes found in multiple isolates were only counted once in the analysis [34].

Microsatellite genotypes of the isolates were determined based on a combination of the allelic data of the 10 loci. The relationships between the genotypes were determined by eBURST analysis [35].

## Results and discussion

In the Republic of Korea, an extensive malaria chemoprophylaxis campaign using chloroquine and primaquine has been conducted annually since the year 1997. The cumulative numbers of the soldiers receiving this treatment exceeded approximately 1.8 million by 2011. Although this chemoprophylaxis has contributed to the containment of vivax malaria, the possibility of the emergence of chloroquine-resistant *P. vivax* strains has been a concern. Indeed, chemoprophylaxis failure has been reported in several cases, despite the attainment of sufficiently high plasma concentrations of hydroxychloroquine [6, 16]. Therefore, monitoring for chloroquine-resistant *P. vivax* is important for the control of malaria in Republic of Korea.

In vitro assays provide drug susceptibility estimates free from the effects of most factors that affect in vivo assays. However, the lack of a robust, standardized and widely applicable protocol for long-term in vitro culture hinders *P. vivax* malaria research. Therefore, short-term ex vivo assays have been successfully used to monitor the chloroquine resistance of *P. vivax* isolates [25, 36]. In addition to the ex vivo chloroquine susceptibility assays, molecular markers, such as *pvmdr1*, have been used to examine chloroquine resistance in *P. vivax* isolates. The *pvmdr1* gene encoding for an ATP binding cassette transporter, has been shown to modulate the responses of *P. vivax* to chloroquine and other anti-malarial drugs [17, 28]. Therefore, the SNPs of *pvmdr1* were evaluated in isolates obtained from patients residing in the demilitarized zone. Compared to the *pvmdr1* gene of the reference *P. vivax* Sal I strain, SNPs at four loci of *pvmdr1*, one non-synonymous mutation (F1076L) and three synonymous SNPs (T529, E1233, and S1358; Table 2) were detected in the *P*. *vivax* isolated from patients. Mutant alleles at position 1076 (F1076L) were present in all isolates (100 %) obtained from samples collected in 2011 and 2012. The number of SNPs at position 529 (T529) and 1233 (E1233) was higher in the 2011 isolates (T529, 57.1 %; E1233, 42.9 %) than in 2012 (T529, 55.6 %; E1233, 33.3 %). Among these four SNPs, F1076L, T529, and E1233 have been reported previously [25]. The newly identified SNP was in codon 1358 (S1358). The S1358 SNP has been reported to be associated with the low chloroquine susceptibility of *P*. *vivax* in Thailand and Myanmar: this result could be considered to be an early sign of the increasing presence of the chloroquine-resistant *P. vivax* strains in the Republic of Korea [25]. However, none of the analysed samples harboured a mutation at codon 976 (Y976F), which has been identified as a possible genetic marker of chloroquine resistance in Southeast Asia and Papua New Guinea [28, 37–39]. This data suggests that chloroquine-resistant *P*. *vivax* may not currently be prevalent in the Republic of Korea. The dN/dS ratios, the ratio of the rate of non-synonymous substitutions to the rate of synonymous substitutions, were 0.935 (29/31) and 0.865 (32/37) for the isolates from 2011 and 2012, respectively. Although the dN/dS ratio of samples collected is below 1 and less than the dN/dS ratio of 2011 samples, these data cannot be concluded that the 2012 samples were under selective pressure.

**Table 2:** Distribution of *pvmdr1* mutations among the *P. vivax* isolates obtained from the South Korean soldiers

Mutation	Year % of mutated isolates (no. of isolates with mutation/total no. of isolates)	
	2011	2012
S513R (AGT/AGA)	0 (0/28)	0 (0/27)
T529 (ACA/ACG)	57.1 (16/28)	55.6 (15/27)
Y976F (TAC/TTC)	0 (0/28)	0 (0/27)
K997R (AAG/AGG)	0 (0/28)	0 (0/27)
F1076L (TTT/CTT)	100 (28/28)	100 (27/27)
E1233 (GAG/GAA)	42.9 (12/28)	33.3 (9/27)
S1358 (TCC/TCT)	0 (0/28)	3.7 (1/27)
K1393 N (AAG/AAC)	0 (0/28)	0 (0/27)
E1396 (GAG/GAA)	0 (0/28)	0 (0/27)

Following the identification of SNPs, the multiple clone infection pattern, genetic diversity as well as inter- and intra-population differences between the *pvmdr1* groups was evaluated using 10 loci. Different alleles sizes observed in a single locus were classified as a multiplicity of infection (MOI). The MOI referred to multiple clone infection. Multiple clone infection was observed in some of the microsatellite loci in 24 of the 55 isolates (49.1 %). Multiple clone infection occurred more frequently in samples from 2011 (60.7 %, n = 17) than in those from 2012 (37.0 %, n = 10). The number of MOI loci per sample was also examined. The highest number of MOI loci per isolate was four, which we observed in a single isolate.

The major alleles of each locus were used for our population genetic analysis. As shown in Table 3, genetic diversity in *P*. *vivax* isolates from 2012 (A = 6.49 ± 0.49, *He* = 0.72 ± 0.14) was greater than in those from 2011 (A = 5.23 ± 0.76, *He* = 0.52 ± 0.29). Moreover, the level of multilocus LD (*I*_*A*_^*S*^) was calculated using allelic data from all *P. vivax* isolates (Table 3). No significant multilocus LD (*I*_*A*_^*S*^ = 0.040, *P* > 0.05) was observed in the *P. vivax* population. Notably, there was greater multilocus LD in the 2012 population (*I*_*A*_^*S*^ = 0.052) than in the 2011 population (*I*_*A*_^*S*^ = 0.028). The genetic diversity of *P. vivax* population in Korea was greater than that reported by Iwagami et al. [26, 27]. Although the sample size (n) was small, the increased genetic diversity and decreased multilocus LD levels of recent *P. vivax* isolates observed appears to be a trend in Korea [26, 40]. The multilocus LD levels were very low (*I*_*A*_^*S*^ = 0.028 in 2011; *I*_*A*_^*S*^ = 0.052 in 2012), suggesting a large possibility of outbreeding between different genotypes. These results are noteworthy since the number of vivax malaria cases is declining in Korea (1772 cases in 2010, 826 cases in 2011, and 542 cases in 2012), which suggests that genetically new *P. vivax* isolates may emerge in the Republic of Korea every year. However, because the isolates exhibited few genetic differences, it would be difficult to conclude that the new *P. vivax* isolates are from different high-risk areas of the Republic of Korea [41].

**Table 3:** Genetic diversity and multilocus linkage disequilibrium in *P. vivax* populations

Year	A	He	*I*_*A*_^*S*^
2011	5.23 ± 0.76	0.52 ± 0.29	0.028
2012	6.49 ± 0.49	0.72 ± 0.14	0.052

*A* average number of the alleles ± SE, *He* average expected heterozygosity ± SE

**Fig. 2:**
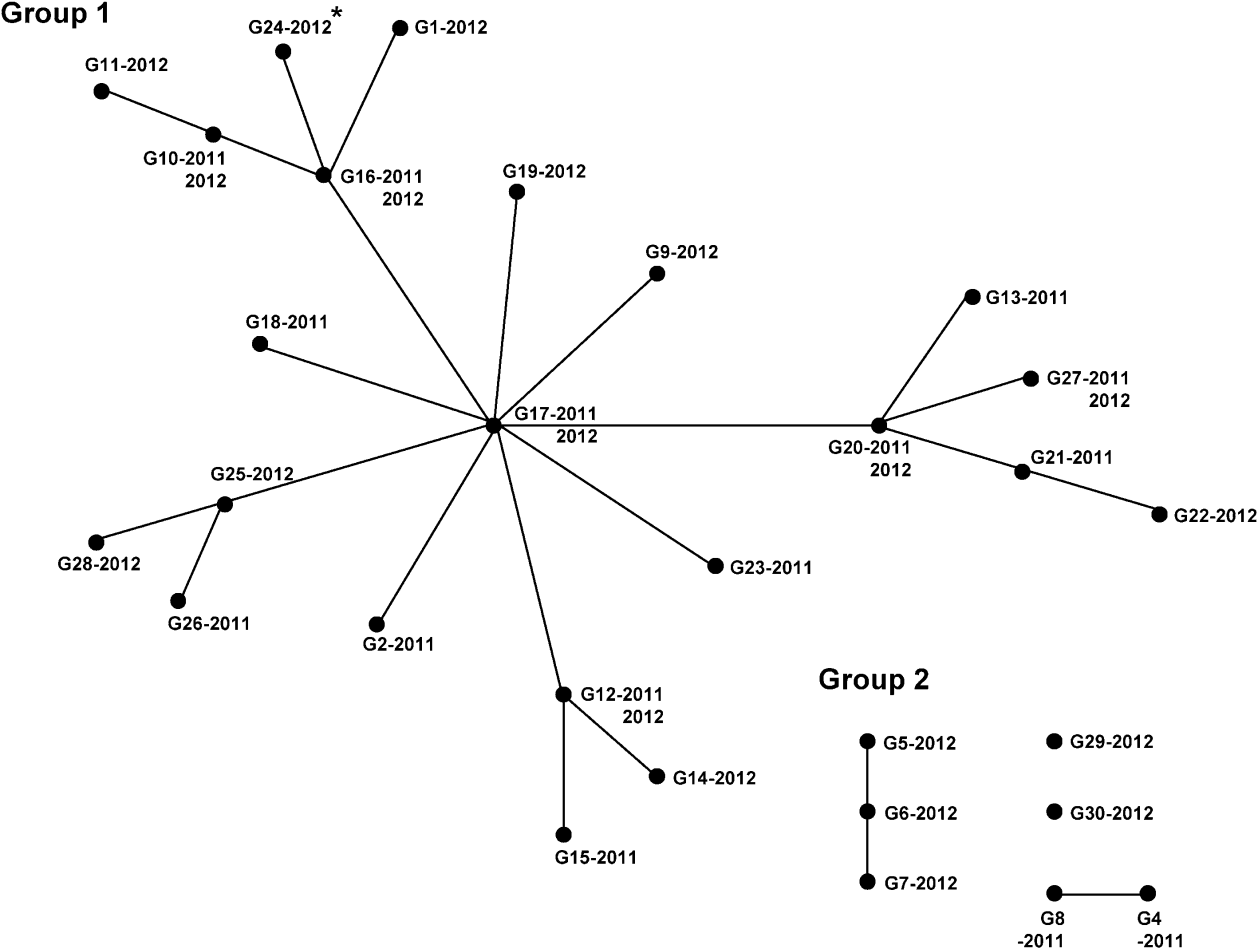
Population structure of the 30 genotypes (n = 55) of *P. vivax* in Republic of Korea were analysed by eBURST. H1-H30 are the microsatellite genotypes. *Genotype includes the newly identified SNP on codon S1358 (TCC/TCT)

Microsatellite genotypes of the 55 isolates were determined based on a combination of the allelic data of the 10 microsatellite loci, and 30 genotypes (G1–G30; Table 4) were identified. Three major genotypes, G12, G16, and G17 were identified. Seven (12.7 %) of the 55 isolates belonged to genotype G16, whereas five isolates each belonged to genotypes G12 and G17. The relationship between the 30 genotypes was determined by eBURST analysis with the following criterion: when two isolates shared more than two identical loci (out of the three loci), these were connected with a branch (Fig. 2). The eBURST analysis revealed two major groups, Group 1 and Group 2. Group 1 contained 22 isolates (40.0 %), including the isolates of genotypes G12, G16, and G17. Group 2 contained three isolates (5.5 %), including the isolates of genotypes G5, G6, and G7. Five isolates of genotypes G4, G8, G29, and G30 were not included in the two major groups, nor were they connected to any other genotypes. Additionally, a single isolate with a new SNP at codon 1358 (S1358) was classified as genotype G24 and was located at the end of the branch, indicating that this genotype is newly introduced into the Republic of Korea *P. vivax* population.

**Table 4:** Thirty genotypes of the *P. vivax* population in the Republic of Korea

	SNP of *pvmdr1*		Total
	2011	2012	
G1		3	3
G2	1		1
G3	1		1
G4	1		1
G5		1	1
G6		1	1
G7		1	1
G8	1		1
G9		1	1
G10	1	1	2
G11		1	1
G12	3	2	5
G13	3		3
G14		1	1
G15	1		1
G16	4	3	7
G17	4	1	5
G18	3		3
G19	2		2
G20	1	1	2
G21	1		1
G22		1	1
G23	1		1
G24^a^		1	1
G25		2	2
G26	1		1
G27	1	1	2
G28		1	1
G29		1	1
G30		1	1
Total	28	27	55

^a^Genotype includes the newly identified SNP on codon S1358 (TCC/TCT)

## Conclusions

In conclusion, the *pvmdr1* gene was analysed in samples collected from South Korean soldiers. The results showed that an isolate with a new SNP (S1358) of *pvmdr1* has been introduced into the Korean *P. vivax* population and that the genetic diversity of the Korean *P. vivax* population is likely to be greater in 2012 than in 2011. Therefore, further continuous monitoring for the presence of chloroquine resistant parasites using molecular markers is needed for the control of vivax malaria in the Republic of Korea.
